# The proposed role of MSL-lncRNAs in causing sex lability of female poplars

**DOI:** 10.1093/hr/uhad042

**Published:** 2023-03-13

**Authors:** Jinyan Mao, Suyun Wei, Yingnan Chen, Yonghua Yang, Tongming Yin

**Affiliations:** State Key Laboratory for Tree Genetics and Breeding, Co-Innovation Center for Sustainable Forestry in Southern China, Key Laboratory of Tree Genetics and Biotechnology of Educational Department of China, Key Laboratory of Tree Genetics and Breeding of Jiangsu Province, Nanjing Forestry University, Nanjing, 210037, China; State Key Laboratory for Tree Genetics and Breeding, Co-Innovation Center for Sustainable Forestry in Southern China, Key Laboratory of Tree Genetics and Biotechnology of Educational Department of China, Key Laboratory of Tree Genetics and Breeding of Jiangsu Province, Nanjing Forestry University, Nanjing, 210037, China; State Key Laboratory for Tree Genetics and Breeding, Co-Innovation Center for Sustainable Forestry in Southern China, Key Laboratory of Tree Genetics and Biotechnology of Educational Department of China, Key Laboratory of Tree Genetics and Breeding of Jiangsu Province, Nanjing Forestry University, Nanjing, 210037, China; Institute for Plant Molecular Biology, State Key Laboratory of Pharmaceutical Biotechnology, School of Life Sciences, Nanjing University, Nanjing 210023, China; State Key Laboratory for Tree Genetics and Breeding, Co-Innovation Center for Sustainable Forestry in Southern China, Key Laboratory of Tree Genetics and Biotechnology of Educational Department of China, Key Laboratory of Tree Genetics and Breeding of Jiangsu Province, Nanjing Forestry University, Nanjing, 210037, China

## Abstract

Labile sex expression is frequently observed in dioecious plants, but the underlying genetic mechanism remains largely unknown. Sex plasticity is also observed in many *Populus* species. Here we carried out a systematic study on a maleness-promoting gene, *MSL*, detected in the *Populus deltoides* genome. Our results showed that both strands of *MSL* contained multiple *cis*-activating elements, which generated long non-coding RNAs (lncRNAs) promoting maleness. Although female *P. deltoides* did not have the male-specific *MSL* gene, a large number of partial sequences with high sequence similarity to this gene were detected in the female poplar genome. Based on sequence alignment, the *MSL* sequence could be divided into three partial sequences, and heterologous expression of these partial sequences in *Arabidopsis* confirmed that they could promote maleness. Since activation of the *MSL* sequences can only result in female sex lability, we propose that MSL-lncRNAs might play a role in causing sex lability of female poplars.

## Introduction

Flowering plants are remarkably diverse in their sexual reproduction, from hermaphrodite to dioecious, with multiple types of transitions of sex morphs, including monoecious, gynomonoecious, andromonoecious, gynodioecious, and androdioecious [1]. About 30% of angiosperms produce unisexual instead of bisexual (hermaphrodite) flowers [2, 3]. The different plant sex morphs are named based on the distribution of bisexual and/or unisexual flowers on plants in a population [1, 4, 5]. Except for the diverse sex morphs, sexual plasticity, such as shifts between sexes, formation of opposite sexual flowers on typically male or female plants, and conversions of unisexual to hermaphrodite flowers, is frequently observed within species and even within individual plants over time [6]. In Ehlers and Bataillon’s study [7], their literature survey found 32 dioecious species (representing 21 different families) exhibiting inconstant sex (termed ‘subdioecious’), in which sex-inconstant plants accounted for at least 5% of individuals in at least one study population.

*Populus* species are normally dioecious, with the alternate sexes of plants baring catkins consisting of staminate (male) or pistillate (female) florets. Labile expression of sex was frequently reported by empirical observation in a variety of *Populus* species [8–10]. In sex-labile poplars, individual trees having both male and female catkins, as well as the same catkins bearing both sexes of florets (monoecious), were observed. Occasionally, perfect florets (hermaphrodite flowers with both sexual organs) presented, but these were very rare [11]. In a review by Delph and Wolf [12], they found that, in dioecious plants, most often it was the male morph that showed labile sex expression. On the contrary, early findings in *Populus* species reported greater lability in the female than in the male sex [11, 13, 14]. Recently, a long-term study of a subdioecious *Populus* × *canescens* family reported that 30% of the progeny expressed labile sexes over their life course. Based on the sex performance of progeny in a number of hybridized families, genetically, the authors only observed inconsistent sex with the female trees, and none of the males in the investigated families showed labile sex or a labile sex phenotype [10]. However, an intensive survey of sex expression in *Populus tremuloides* showed no significant differences in labile sex expression between the male and female trees [15]. Therefore, both sexes of poplars expressed labile sex, but whether the female expressed greater labile sex than the male remained controversial. For dioecious plants, the emergence of intermediate sex morphs was due to either male or female lability. However, whether the individual with inconsistent sex had transitioned from being a male or female tree  can only be judged based on the degree to which the sex-labile individual looked more like a male or a female in the early empirical studies. However, judgment merely based on phenotype is problematic.

Although profound efforts have been exerted in the study of sex lability in flowering plants [6, 7, 10], earlier studies mainly addressed the phenotypic performance of sex lability. The molecular mechanism underlying plants’ sex lability remains largely unknown. In recent years, the identification of sex determination genes in a variety of dioecious plants, such as persimmon [16], garden asparagus [17], kiwifruit [18, 19], poplars [20, 21], and others, has offered a great opportunity to explore the molecular mechanism underlying sex lability in plants. In the aforementioned dioecious plants, different genes were found to be involved in sex separation of the affected plants, but they functioned through a similar molecular mechanism, i.e. a Y-specific gene duplicated from an autosomal female gene evolved into a female suppressor, which generated siRNAs to block the expression of the female gene [16–21]. Therefore, it is reasonable that lower expression of the female suppressor in the male would cause male sex lability. The female suppressor functions through the siRNA pathway [16–21]. It is known that lncRNAs are transcribed in a developmentally regulated manner or as a response to external stimuli [22–24], which is consistent with the phenomena that labile sex expression is greatly affected by the developmental stage of the plants [6, 19] and environmental factors [6, 10]. However, in plants with the XY sex determination system, the female sex would not be affected by the Y-specific female suppressor. Thus, different genetic factors should be involved in labile sex expression of females, but little is known on this aspect thus far.

For unisexual flower development, four stages of sexual organ abortion are recognized: before the initiation of stamen or carpel primordia (stage 0); early in stamen or carpel development (stage 1); pre-meiosis (stage 2); and post-meiosis (stage 3) [25]. Sexual organ abortions occurring at stage 1, stage 2, and stage 3 would form flowers bearing apparently opposite sexual organs, leading to the emergence of cryptic dioecious plants, such as persimmon, garden asparagus, and kiwifruit. Poplars are complete dioecious species, whose male and female flower primordia differentiate before the initiation of stamens and pistils. Thus, genes triggering separation of poplar sexes should be expressed in the very early stage of flower development. A large number of sexually differentially expressed genes have been detected between male and female flowers of poplars. However, most of the sex-limited and sex-biased genes do not reside on sex chromosomes [26–28]. Since the flower samples analyzed in most previous studies were flushed and at the late developmental stages, the detected sex-limited and sex-biased genes might only be involved in the development of male or female flower organs, whereas genes triggering sexual differentiation of poplar flower primordia should function before or at the early stage of stamen and pistil initiation.

Our previous study on *Populus deltoides* revealed that that the male trees harbored two Y-specific genes, *FERR-R* and *MSL* [21], which determined the differentiation of opposite sexual flower primordia. *FERR-R* functions as a female suppressor to block the expression of the female gene, *FERR*, whereas *MSL* functions as a male promoter. Heterologous transformation of *MSL* sequences in *Arabidopsis* showed that they could promote the development of androecia [21]. It was noteworthy that female poplars did not possess the male-specific *MSL*, but a large number of partial sequences with high sequence similarity to *MSL* (referred as *MSL*-homologous sequences hereafter) were found to be scattered in the poplar genome. Results of this study showed that activated transcription of the *MSL*-homologous sequences could promote maleness in *Arabidopsis*. If these maleness promoters were expressed in female poplars, they would lead to female lability. This study provides a unique perspective for better understanding of female sex lability in poplars.

## Results

### Y-specific *MSL* and *MSL*-homologous sequences in the poplar genome

**Figure 1:**
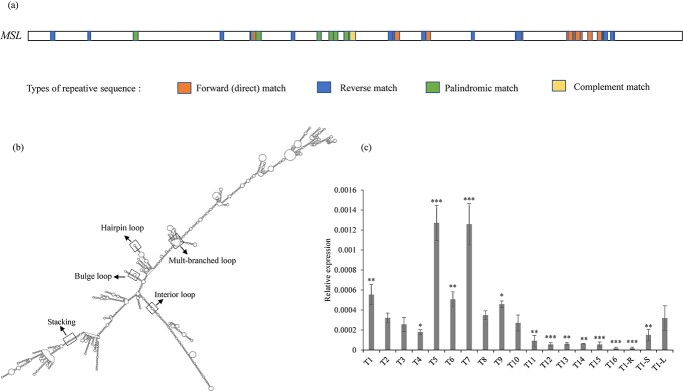
Sequence and structural characteristics of *MSL* and its expression in different developmental stages of male flower buds. (a) Repetitive sequences in *MSL*. (b) Secondary structure prediction. (c) Relative expression of *MSL* in male flower buds sampled at T1–T16 (see Materials and methods section) and in root, stem, and leaf tissues; T1-R, T1-S, and T1-L represent root, stem, and leaf, respectively, sampled at T1. Data represent means from three replicates, with error bars indicating standard deviation. Asterisks at the top of bars indicate statistically significant differences between leaf and other tissues with a *P*-value ≤.05 (^*^), .01 (^**^) or .001 (^***^).

*MSL* was a male-specific gene detected in *P. deltoides*. Based on sequence annotation, it was identified as a transposable element in the LTR/Gypsy transposon family, with a total length of 2868 bp [21]. Our sequence analysis showed that this gene was rich in repetitive sequences, mainly including forward matches, reverse matches, complement matches, and palindromic matches (Fig. 1a). Secondary structure prediction for *MSL* detected high frequencies of stacking, bulge loop, interior loop, hairpin loop, and multi-branched loop (Fig. 1b), and contents of these structural characteristics indicated that *MSL* was a typical sequence generating lncRNAs (Table 1). We further analyzed the lncRNA-seq data of male and female flower buds at five different times (Supplementary Data Fig. S1a–e). Results showed that the Y-specific *MSL* produced transcripts across different developmental stages of the flower tissue. Examination of transcription for the forward and reverse strands of *MSL* revealed that both strands produced transcripts, with the active transcription regions varying across the different sampling times (Supplementary Data Fig. S1f–j).

**Table 1:** Contents (%) of stacking, bulge loop, interior loop, hairpin loop, and multi-branched loop in the predicted secondary structure of *MSL*.

**Structural characteristic**	**Content in lncRNA** ^**a**^	**Content in *MSL***	**Content in *MSL-1***	**Content in *MSL-2***	**Content in *MSL-3***
Stacking	5–10	8.11	7.53	11.12	6.71
Bulge loop	1–4	2.7	3.42	3.74	4.19
Interior loop	2–8	3.66	2.28	2.72	3.09
Hairpin loop	0.6–3.6	2.4	2.63	3.0	2.58
Multi-branched loop	0.9–5.4	4.21	5.59	4.37	3.74

a Refer to Li [29].

**Figure 2:**
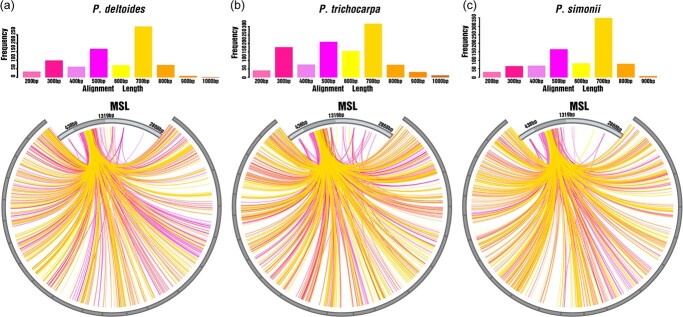
Alignment of the partial homologous sequences (≥200 bp) with *MSL* in different poplar genomes. (a) *P. deltoides*. (b) *P. trichocarpa*. (c) *P. simonii*. The *x*-axis shows alignment length and the *y*-axis shows the frequency of alignment lengths. In each chart, the circle displays the distribution of *MSL*-homologous sequences on each chromosome. The chromosomes are arranged along the circle from the right end to the left end.

Alignment analysis with the sequence of *MSL* identified a large number of partial *MSL*-homologous sequences (≥200 bp) in the genomes of a variety of sequenced poplars, including *P. deltoides*, *P. alba*, *P. davidiana*, *P. trichocarpa*, *P. davidiana* × *P. bolleana*, *P. tomentosa*, *P. simonii*, *P. ilicifolia*, *P. euphratica*, *P. alba* × *P. glandulosa*, and *P. sibirica* (Fig. 2, Supplementary Data Fig. S2), and many of them resided on the Y chromosome (Supplementary Data Fig. S3). In *P. deltoides*, a total of 1149 partial *MSL*-homologous sequences (≥200 bp) were detected, which were scattered along the 19 poplar chromosomes and were abundant on chromosomes I and XIX (Supplementary Data Fig. S4). Since the quality of the analyzed genome assemblies varied markedly, the numbers of partial sequences (≥200 bp) with homology to *MSL* varied greatly in different poplar genome assemblies. Nevertheless, based on the abundance of the *MSL*-homologous sequences, the *MSL* gene can be divided into three parts: *MSL-1* covers 438 bp from the 3′ end, *MSL-2* spans 881 bp in the middle, and *MSL-3* encompasses 1549 bp of the 5′ end (Fig. 2, Supplementary Data Fig. S2). In poplar genomes, the homologous sequences of *MSL-1* and *MSL-3* are relatively scarce, while the homologous sequences of *MSL-2* are very abundant.

### Both strands of *MSL* promote maleness and possess multiple *cis*-acting elements

**Figure 3:**
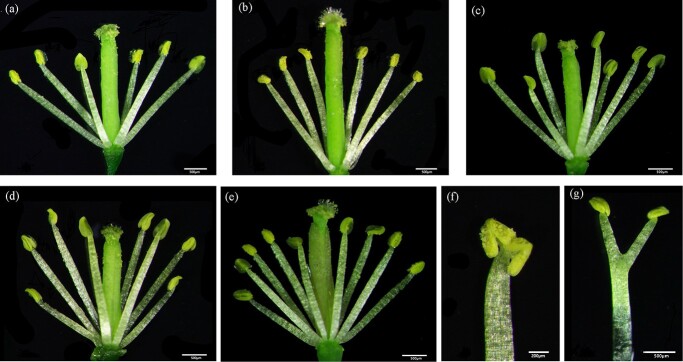
Floral characteristics of *A. thaliana* wild-type and transgenic plants. (a) Characteristics of wild-type *Arabidopsis* flowers. The flowers of wild-type *Arabidopsis* have tetradynamous stamens (four long and two short stamens). (b–g) Characteristics of transgenic *Arabidopsis* plants. (b) The flower has six long stamens. (c) The flower has seven stamens. (d) The flower has eight stamens. (e) The flower has nine stamens. (f) The flower has two anthers on one filament. (g) The flower has branched stamens.

Overexpression of the forward strand of *MSL* in *Arabidopsis* demonstrated that it functioned as a maleness promoter [21]. In this study, we constructed a transformation of the reverse strand of *MSL* with the CaMV35S promoter. Overexpression of the reverse strand of *MSL* in *Arabidopsis* exhibited the same phenotypic changes in the flower sexual organs as those of the forward strand [21]. In contrast to the tetradynamous stamens (four long and two short ones) of the wild-type flowers, we observed flowers with six long stamens, seven, eight, or nine stamens, or stamens bearing two anthers, or branched stamens in the transformed lines (Fig. 3). Unlike its effect on androecia, no visible changes were observed with gynoecia between the wild-type and transformed lines. Therefore, function to promote maleness was evident for both forward and reverse strands of *MSL*.

The type of transcription factor that could induce lncRNA transcription has not been well identified [30]. The majority of lncRNAs have been shown to be transcribed through RNA polymerase II, although some lncRNAs are generated by RNA polymerase III [31–33]. Multiple *cis*-activating elements (TATA box, CAAT box, transcription initiators, and response element) were observed on both strands of *MSL* (Supplementary Data Fig. S5a). Thus, transcription of lncRNAs from both strands of *MSL* might be modulated by the inner *cis*-acting elements. Therefore, we constructed transformation vectors with the positive strand of *MSL* without the CaMV35S promoter. A total of 27 transformation lines were identified by PCR amplification and β-glucuronidase (GUS) staining (Supplementary Data Fig. S5b and c). The quantitative real-time PCR (qRT–PCR) assay detected expression of *MSL* in all of the transformed lines (Supplementary Data Fig. S6), and we observed promotion of androecia similar to that seen  in the transformed lines driven by the CaMV35S promoter (Supplementary Data Fig. S7), indicating that transcription of *MSL* could be activated by the inner *cis*-acting elements.

### The partial *MSL* sequences could function to promote maleness

As described above, we divided the *MSL* sequence into three portions, and structural predication of *MSL-1*, *MSL-2*, and *MSL-3* showed that these partial sequences could generate lncRNAs (Table 1, Supplementary Data Fig. S8). A trace amount of lncRNAs transcribed by *MSL*-homologous sequences was also detected in normal females. Transformation constructs were built with *MSL-1*, *MSL-2*, and *MSL-3*, and the CaMV 35S promoter. A total of 50 overexpression lines were identified. Examined with a stereoscopic microscope, we observed flowers with seven stamens, or eight stamens, or branched stamens, or stamens bearing multiple anthers (Supplementary Data Fig. S9a) in transgenic plants of *MSL-1*, whereas in transgenic plants of *MSL-2*, and *MSL-3* similar phenotypes of abnormal stamens were observed (Supplementary Data Fig. S9b and c). By contrast, no visible changes in pistil development were detected. Therefore, a transgenic study on *MSL-1*, *MSL-2*, and *MSL-3* showed that these partial sequences could function as the maleness promoters. By using *ACTIN2* as the internal reference, qRT–PCR assay showed that, in general, the relative expression level was *MSL-3* > *MSL-2* > *MSL-1* (Supplementary Data Fig. S10).

### Genes affected by *MSL* in transgenic *Arabidopsis*

No homologous sequences of *MSL* were detected either in the *Arabidopsis* genome or in the other genomes under investigation, including, *Oryza sativa*, *Zea mays*, *Amborella trichopoda*, *Vitis vinifera*, *Asparagus officinalis*, *Diospyros lotus*, and *Carica papaya*. Heterologous expression of the *MSL* sequences could promote the development of androecia in transgenic *Arabidopsis*. With the overexpression lines of the *MSL* forward strand, we analyzed the affected genes in transgenic *Arabidopsis* by transcriptomic sequencing. In comparison with the wild-type *Arabidopsis*, we identified 28, 52, and 21 differentially expressed genes (DEGs) in *Arabidopsis* flowers at T1, T2, and T3 stages, respectively. Among them, 15 DEGs were shared across the three stages, 2 DEGs were shared between T2 and T3, and the others were differentially expressed only in T1, T2, or T3 (Supplementary Data Fig. S11). For the detected DEGs, most of them (~87%) were found to be upregulated. We further analyzed the interaction between MSL-lncRNAs and miRNAs. Twenty miRNAs were predicted to interact with the MSL-lncRNAs, but none of the detected DEGs were the target genes of these miRNAs, indicating that *MSL* might not act as the miRNA sponge to regulate expression of the affected genes.

For the 15 DEGs shared across different developmental stages, all were upregulated in the transgenic lines (SupplementaryData Fig. S12). Analyzing the conserved domains in these DEGs revealed that three genes (*AT1G80960.4*, *AT4G23580.1*, and *AT2G02240.1*) contained the F-box domain. The F-box family proteins were reported to be involved in regulating the development of floral meristems and floral organs [34–36], e.g. the F-box *UFO* gene was revealed to interact with and upregulate *ASK1* and *LEAFY* in Class B [37]. According to the ABC model underlying flower development, stamen development was jointly determined by Class B and Class C genes, among which Class B genes only affected stamens and did not affect pistils. Among the 15 DEGs, it was noteworthy that *AT4G22440.1* exhibited the highest constitutive expression in the transgenic lines, while no expression of this gene was detected in flowers of wild-type *Arabidopsis* across different developmental stages (Supplementary Data Fig. S12). Functional annotation of *AT4G22440.1* showed that it was a gene of unknown function. Although we could not pinpoint the genes that directly interacted with *MSL* with the current data, the transcriptomic analysis detected a list of candidate genes for further exploration in future studies.

## Discussion

lncRNAs are tentatively defined as RNA molecules >200 nucleotides long that do not encode proteins [22–24]. Genome-wide studies indicate the existence of a large number of lncRNAs in eukaryotic genomes. However, only limited numbers of functional lncRNAs have been identified thus far. Nonetheless, the immense regulatory potential of lncRNAs is already evident [22–24]. Our results showed that the poplar MSL-lncRNAs promoted androecium development in heterologous transgenic lines of *Arabidopsis*. Some studies also showed that lncRNAs played an important role in animal and plant reproduction [32, 38–45]. Here we performed a systematic study on different aspects of the *MSL* gene that generated lncRNAs. Our results showed that both strands of *MSL* function, and transcription of *MSL* can be activated by the inner *cis*-acting elements. Based on sequence alignment, the *MSL* sequence could be divided into three portions, and each partial sequence could function as the maleness promoter. Sex lability occurs in either male or female trees. In subdioecious individuals (bearing female and male flowers on the same tree) of *Populus lasiocarpa*, which were genetically identified as males (possessing the Y-specific *FERR-R* and *MSL*), lower expression of *FERR-R* resulting in lower methylation of *FERR* was detected (unpublished data). Thus lower expression of *FERR-R* in sex-labile males than in normal males will lead to male sex lability. Female poplars do not possess the Y-specific *FERR-R* and *MSL*. This showed that knockout of *FERR* (termed *ARR17*) in female *Populus tremula* would result in complete conversion of female to male [20]. Therefore, female lability of poplars might be caused by partial reduction of *ARR17* expression for an unknown reason. But if *ARR17* itself triggers female lability, the co-sexual locus should be co-located with the *ARR17* gene. In Sabatti *et al*.’s study [10], the co-sexual locus underlying female lability was mapped to a position other than *ARR17*, indicating that female lability should relate to genetic factors other than *ARR17*.

**Figure 4:**
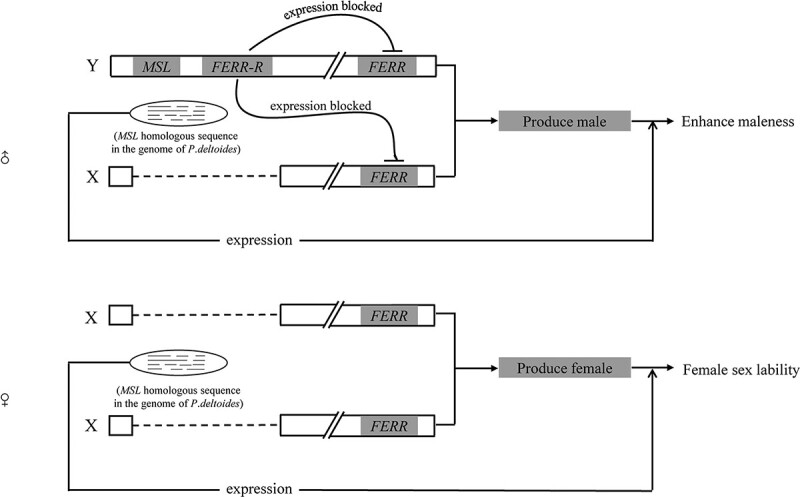
A proposed genetic model showing the role of MSL-lncRNAs in causing female sex lability in poplars. There is a large number of *MSL*-homologous sequences in the poplar genome. Only trace amounts of lncRNAs transcribed by *MSL*-homologous sequences were detected in normal females. Heterologous expression of MSL-lncRNAs can promote maleness in *Arabidopsis*, and thus we propose that increased transcription of MSL-lncRNAs would lead to sex lability of female poplar. In this model, the male sex is not affected because MSL-lncRNAs function as maleness promoters.

We detected a large number of *MSL*-homologous sequences in the poplar genome. Heterologous expression of MSL-lncRNAs can promote maleness in *Arabidopsis*, and thus we proposed that activated transcription of MSL-lncRNAs would lead to sex lability of female poplar. The *MSL* sequences function through the lncRNA pathway. lncRNAs are normally expressed at low levels, can interact with DNA, RNA, and proteins directly or indirectly in eukaryotes, and with significant molecular function [44, 46–48]. This class of RNAs was transcribed in a developmentally regulated manner or as a response to external stimuli [22–24]. Some poplars express labile sex in particular developmental stages and sex lability was greatly affected by environmental factors [6, 10]. Thus, the hypothesis that female sex lability of poplars might be modulated through the lncRNA pathway is highly possible. Based on the effect of *MSL* sequences on androecium development observed in this study, we proposed a molecular model triggering sex lability in female poplars. In this model, activated transcription of *MSL*-homologous sequences only leads to female lability, but does not cause male lability (Fig. 4).

Scaling to the expression of the internal reference gene, *MSL* sequences were expressed at a high level in transgenic *Arabidopsis* (Supplementary Data Figs S6and S10). By contrast, very low expression of *MSL* was detected in poplar (Fig. 1c). Whether there is an outburst of *MSL* transcription in the very early development stage of poplar flowers needs to be explored in the meristem cells of flower primordia. Moreover, to elucidate whether overexpression of MSL-lncRNAs would lead to sex shifting in poplars, transformation studies need to be performed on female poplars. Thus, we still lack experimental proof in these aspects. There might be some other genetic factors that could cause sex lability in female poplars. Nevertheless, heterologous transformation showed that MSL-lncRNAs can promote maleness. It is reasonable to propose that they might play a role in causing labile sex expression in female poplars.

## Materials and methods

### Analyzing *MSL* and its homologous sequences

The repetitive sequences in *MSL* (Supplementary Data S1) were analyzed by using REPuter [49]. Parameters were as follows: Match Direction choose Forward (direct)[−f], Reverse[−r], Complement[−c] Palindromic[−p]; Hamming Distance was 3; Minimal Repeat Size was 8; Maximum Computed Repeats was 50. The predicted secondary structures of *MSL* sequences were built by using the minimum free energy (MFE) method of RNAfold [50], in which the component ratios of stacking, bulge loop, interior loop, hairpin loop, and multi-branched loop were calculated based on structural predictions built with the dot-bracket notation method of RNAfold [29]. Sequence alignment analysis was conducted with SeqHunter software [51], with an E-value of 10^−5^, and results were visualized by using RStudio software [52] and Circos [53]. To detect the *MSL*-homologous sequences, we analyzed 11 poplar genomic sequence assemblies, among which the genomic sequences of *P. deltoides* and *P. davidiana* × *P. bolleana* were our in-house databases, and those of *P. alba* (PRJNA638679), *P. davidiana* (PRJNA628187), *P. trichocarpa* (PRJNA17973), *P. tomentosa* (PRJNA613008), *P. simonii* (PRJNA545814), *P. ilicifolia* (PRJNA471950), *P. euphratica* (PRJNA268063), *P. alba* × *P. glandulosa* (PRJNA526157), and *P. sibirica* (https://www.ncbi.nlm.nih.gov/genome/104514) were the online databases from NCBI (https://www.ncbi.nlm.nih.gov/genome/?term=populus). The distribution of *MSL*-homologous sequences along each poplar chromosome was plotted by using MG2C v2.1 [54]. The *cis*-acting elements of *MSL* sequences were identified by using PlatCARE [55].

### Heterologous expression of *MSL* sequences in *Arabidopsis*

The DNA sequences of *MSL*, *MSL-1*, *MSL-2*, and *MSL-3* were separately cloned into the binary vector of p2301-35S plus to build the transgenic constructs. To create constructs that did not possess the CaMV 35S promoter, the 35S promoter was removed by the Sse8387I and XbaI restriction endonucleases (Takara, Biotech Co. Ltd, Nanjing, China), and the processed vectors were purified using the TIANgel Purification Kit (Tiangen Co. Ltd, Nanjing, China). The vector constructions were transformed into Trans1-T1 Chemically Competent Cells (TransGen Biotech Co. Ltd, Nanjing, China). Positive clones were selected on LB-Kan^+^ agar plates and verified by PCR. Vectors in positive clones were extracted and transformed into *Agrobacterium tumefaciens* strain GV3101 (pMP90).

Seeds of *Arabidopsis thaliana* ecotype Columbia-0 (Col-0) were sterilized in ethanol 70% for 30 seconds, washed with sterile H_2_O three times, then soaked in 5% sodium hypochlorite for 10 minutes, and washed with sterile H_2_O six times. Subsequently, seeds were germinated on 1/2 Murashige–Skoog (MS) medium (3% sucrose, 0.6% agar), and kept in a cold room at 4°C for 2 days to break dormancy, and then transferred to a plant culture room with a temperature of 19–23°C, humidity of 65–70%, and a light cycle of 16 hours light/8 hours darkness. After 10 days, seedlings were planted in nutrient soil (peat:perlite:vermiculite = 3:1:1) in a growth chamber under a temperature of 23°C day/15°C night, humidity of 70%, and photoperiod of 16 hours light/8 hours darkness until transformation. Transformation of *Arabidopsis* was conducted following the floral dip method [56], and seeds of transgenic lines were harvested. Sterilization of transgenic seeds was performed as described above. Transgenic plants were selected on 1/2 MS medium containing 50 mg/ml kanamycin. Kanamycin-resistant seedlings were further confirmed by GUS staining and PCR amplification. The wild-type and transgenic plants were grown in a growth chamber for 30 days, then flowers at different developmental stages were sampled and observed under an Olympus SZX10 microscope (Olympus Co. Ltd, Tokyo, Japan).

### Transcription and expression of *MSL* sequences

Male and female flower buds of *P. deltoides* at T2 (18 June), T3 (3 July), T4 (18 July), T5 (3 August), and T8 (1 December) were sampled for chain-specific lncRNA-seq analysis. The flower buds collected on each date were divided into three male repeats and three female repeats, and each repeat was subjected to sequencing separately. The RNAprep Pure Plant Kit (Tiangen Co. Ltd, Nanjing, China) was used to extract RNAs from flower buds and leaf tissues. TransScript One-Step gDNA Removal and cDNA Synthesis SuperMix (TransGen Biotech Co. Ltd, Nanjing, China) were used to reverse-transcribe RNA to cDNA with random primers to establish the lncRNA libraries. Illumina sequencing experiments (Illumina NovaSeq 6000, USA) were performed for strand-specific lncRNA-Seq, as lncRNA-Seq worked for transcripts with and without polyA tails. STAR v2.5.3 [57] was used to map the lncRNA-Seq reads onto the reference, and the results were visualized using IGV v2.4.14 [58].

For the transgenic plants, expression of the *MSL* sequences was measured by qRT–PCR. The rosette leaves of 25-day-old plants of wild-type and transgenic plants were sampled, frozen in liquid nitrogen, and stored at −80°C until RNA extraction. Total RNA was extracted by using an RNA Easy Fast Plant Tissue Kit (Tiangen Co. Ltd, Nanjing, China), and cDNA reverse transcription was conducted by using an lnRcute lncRNA First-Strand cDNA Kit (Tiangen Co. Ltd, Nanjing, China). The qRT–PCR was conducted by using an lnRcute lncRNA qPCR Kit (Tiangen Co. Ltd, Nanjing, China), with the *Actin2* gene as the internal reference. Gene-specific primers for qRT–PCR were designed by Primer Premier 5.0 software (Premier Biosoft International, Palo Alto, California, USA). The qRT–qPCR reaction was performed on a 7500 Fast Real-Time PCR System (Applied Biosystems Co. Ltd, New York, USA), with the following protocol: denatured at 95°C for 3 minutes, then 40 cycles of 95°C for 15 seconds, 60°C for 15 seconds, and 72°C for 30 seconds, followed by the default melt curve. Expression level was quantified using the 2^−ΔΔCt^ method [59]. Each sample was measured with three technical replicates. Data were analyzed using Excel 2010 and SPSS 26.0 software (version 26.0). Significance was tested by one-way ANOVA and Duncan’s test. Similarly, qRT–PCR was conducted with flower buds of a male *P. deltoides* tree. Poplar inflorescences flushed in March 2021, but the floral primordial buds differentiated in early June of last year (2020). The analyzed samples collected on 3 June (T1), 18 June (T2), 3 July (T3), 18 July (T4), 3 August (T5), 18 August (T6), 3 September (T7), 1 December (T8), 15 January (T9), 30 January (T10), 15 February (T11), 1 March (T12), 5 March (T13), 9 March (T14), 15 March (T15), 17 and March (T16), which covered the complete development process of poplar flowers from differentiation till bloom. Leaf and root samples were collected from clonal seedlings of the same tree. The poplar *UBIQUITIN* (*PtUBQ*) gene was used as the internal reference gene, according to Brunner *et al*.’s study [60].

### Analyzing the affected genes in transgenic *Arabidopsis*

To investigate the genes affected by *MSL*, we selected the transgenic lines that overexpressed the forward strand of *MSL*. Transcriptome sequencing was conducted with flower samples collected at T1, T2, and T3 (Supplementary Data Fig. S13) from the third generation of transgenic plants and from wild-type *Arabidopsis*. The developmental stages of flowers were determined as described by Smyth *et al*. [61]. In the transcriptome sequencing, each sample was repeated with three biological replicates. RNA preparation was conducted as described above. Transcriptome sequencing was performed on the Illumina NovaSeq 6000 sequencer (Illumina, CA, USA). Raw reads were processed following the description by Bolger *et al*. [62]. Gene expression was evaluated by mapping the clean reads to *TAIR* 11 (the *A. thaliana* reference genome) by using HISAT2 software [63]. Analysis of the DEGs was conducted with the R packages of DESeq2 [64], with threshold of |log2 (Fold Change)| > 2 and false discovery rate <.01.

## Acknowledgements

The work was supported by the National Key Research and Development Plan of China (2021YFD2200202), the Natural Science Foundation of China (32071795), and the Key Research and Development Project of Jiangsu Province, China (BE2021366).

## Author contributions

T.Y. conceived and designed the research. J.M. and Y.C. performed the experiments. S.W. and Y.Y. analyzed the data. T.Y. and J.M. drafted the manuscript.

## Data availability

The genome assembly of *P. deltoides* is available at https://www.ncbi.nlm.nih.gov/bioproject/PRJNA599215 with accession number PRJNA599215, and that of *P. davidiana × P. bolleana* is unpublished. lncRNA-seq data for poplar samples are available at https://www.ncbi.nlm.nih.gov/bioproject/PRJNA599218 with accession number PRJNA599218. RNA-seq data for the *Arabidopsis* samples are available at https://www.ncbi.nlm.nih.gov/bioproject/PRJNA885296 with accession number PRJNA885296.

## Conflict of interest

The authors declare that they have no conflicts of interest.
